# RAD9 deficiency enhances radiation induced bystander DNA damage and transcriptomal response

**DOI:** 10.1186/1748-717X-9-206

**Published:** 2014-09-18

**Authors:** Shanaz A Ghandhi, Brian Ponnaiya, Sunil K Panigrahi, Kevin M Hopkins, Qingping Cui, Tom K Hei, Sally A Amundson, Howard B Lieberman

**Affiliations:** Center for Radiological Research, Columbia University College of Physicians and Surgeons, New York, NY 10032 USA; Department of Environmental Health Sciences, Mailman School of Public Health, Columbia University, New York, NY 10032 USA

**Keywords:** RAD9, Ionizing radiation, Bystander, Gene expression, Micronucleus, Chromosome aberrations

## Abstract

**Background:**

Radiation induced bystander effects are an important component of the overall response of cells to irradiation and are associated with human health risks. The mechanism responsible includes intra-cellular and inter-cellular signaling by which the bystander response is propagated. However, details of the signaling mechanism are not well defined.

**Methods:**

We measured the bystander response of *Mrad9*^*+/+*^ and *Mrad9*^*−/−*^ mouse embryonic stem cells, as well as human H1299 cells with inherent or RNA interference-mediated reduced RAD9 levels after exposure to 1 Gy α particles, by scoring chromosomal aberrations and micronuclei formation, respectively. In addition, we used microarray gene expression analyses to profile the transcriptome of directly irradiated and bystander H1299 cells.

**Results:**

We demonstrated that *Mrad9* null enhances chromatid aberration frequency induced by radiation in bystander mouse embryonic stem cells. In addition, we found that H1299 cells with reduced RAD9 protein levels showed a higher frequency of radiation induced bystander micronuclei formation, compared with parental cells containing inherent levels of RAD9. The enhanced bystander response in human cells was associated with a unique transcriptomic profile. In unirradiated cells, RAD9 reduction broadly affected stress response pathways at the mRNA level; there was reduction in transcript levels corresponding to genes encoding multiple members of the UVA-MAPK and p38MAPK families, such as STAT1 and PARP1, suggesting that these signaling mechanisms may not function optimally when RAD9 is reduced. Using network analysis, we found that differential activation of the SP1 and NUPR1 transcriptional regulators was predicted in directly irradiated and bystander H1299 cells. Transcription factor prediction analysis also implied that HIF1α (Hypoxia induced factor 1 alpha) activation by protein stabilization in irradiated cells could be a negative predictor of the bystander response, suggesting that local hypoxic stress experienced by cells directly exposed to radiation may influence whether or not they will elicit a bystander response in neighboring cells.

**Electronic supplementary material:**

The online version of this article (doi:10.1186/1748-717X-9-206) contains supplementary material, which is available to authorized users.

## Background

The radiation induced bystander effect is the biological response of unirradiated cells in contact with or in the vicinity of cells directly exposed to radiation. This response has been demonstrated using a wide variety of cell types, including primary cells [1], hematopoietic cells [2], cancer cells [3], as well as *in vivo* [4]. Bystander effects have been assessed by multiple radiation-related endpoints such as clonogenic survival, apoptosis, micronuclei formation and DNA damage [3]. There is evidence for inter-cellular bystander signaling mediated by reactive oxygen species [5], cytokines [6], gap-junction proteins [7], and extracellular factors such as TGFβ [8]. A global transcriptomic bystander response involving NFκB has also been described in primary cells [9].

The DNA damage response (DDR) to direct radiation exposure includes a multi-component network of pathways, leading to activation of ATM and ATR kinases that sense structural damage to DNA, further triggering a cascade of events that affect cell fate [10]. The heterotrimeric protein complex made up of RAD9, HUS1 and RAD1 (i.e. 9-1-1) is part of this signaling network, and has numerous functions impacting on the way cells respond to DNA damage, including cell cycle checkpoint control and DNA repair [10–14]. Little is known about the relationship between DNA damage signaling in cells that are directly irradiated and their corresponding unirradiated bystanders. However it is established that ATR upstream to ATM is important for the bystander response [15]. In addition, one component of 9-1-1 has been tested for a role in the bystander response; Cell cycle checkpoint control protein RAD9 influences the cellular response to both direct and bystander radiation exposure. *Mrad9* null mouse embryonic stem cells, relative to *Mrad9*^*+/+*^cells, demonstrate enhanced radiation induced bystander responses, including apoptosis and micronuclei formation [14]. RAD9 has many functions, including regulation of cell cycle checkpoints, DNA repair and the ability to transcriptionally activate downstream target genes [12]. However, it is not known which of the many functions of RAD9 is critical for influencing the bystander response.

In this study, we investigated the role of mouse and human RAD9 protein in the ionizing radiation induced bystander response, by assessing the effects of RAD9 level reduction on acquisition of DNA damage and changes in transcriptomic profiles. We demonstrate that *Mrad9* null, relative to *Mrad9*^*+/+*^, in mouse embryonic stem cells enhances the frequency of direct and bystander radiation induced chromatid aberrations, which persist over multiple cell divisions. In the human non-small cell lung carcinoma cell line H1299, we found that RNA interference-mediated RAD9 reduction increases the frequency of micronuclei formation after direct and bystander ionizing radiation exposure. A significant gene expression response was also detected in these cells. There was a correlation between cells that showed an increase in micronuclei frequency and the gene expression response measured in parallel. Analysis of microarray gene expression data predicted SP1 and NUPR1 transcription factors to be involved in the radiation response of cells where bystander effects were observed. We also predict that HIF1α activation status may be different in directly irradiated cells that generate a bystander response in neighboring cells, compared to those that do not.

## Methods

### Cell culture, protein isolation and Western blotting

An isogenic set of mouse embryonic stem cells, which were either *Mrad9*^*+/+*^*, Mrad9*^*−/−*^*,* or the latter ectopically expressing *Mrad9*^*+*^ [16], were grown at 37°C, 5% CO_2_ in Knockout-DMEM (Invitrogen), with 15% ES cell qualified fetal bovine serum, 0.1 mM non-essential amino acids, 2 mM L-glutamine, 10^−4^ M beta-mercaptoethanol, 100 U/ml penicillin/streptomycin, and 10^−3^ U/ml leukemia inhibitory factor (LIF, available as “ESGRO” from Chemicon). Tissue culture plates and dishes were coated with a 0.1% gelatin solution and used routinely for cell passage and maintenance. Mylar dishes were coated with a 4 mg/ml fibronectin solution (Sigma). Human non-small cell lung carcinoma cells, H1299 (ATCC, CRL-5803), were grown in DMEM containing 10% FBS plus penicillin/streptomycin (50 μg/ml) at 37°C in a humidified 95% air, 5% CO_2_ incubator. H1299 cells were infected with pSUPER.retro.puro viral vector containing a *RAD9* shRNA to promote knockdown of expression as described [17], and grown in medium supplemented with puromycin (2 μg/ml) for selection of stable clones. RAD9 protein levels in cell lysates were analyzed by Western blotting using anti-RAD9 antibody (BD Transduction Laboratories, catalog no. 611324) and anti-beta-actin antibody (Sigma, catalog no. A5316). Clones with greater than 70% reduction in RAD9 level, relative to parental control cells, were chosen for additional analyses.

### Mouse ES cell irradiation and chromosome assay

All irradiations were carried out using confluent cells plated on concentric Mylar dishes as described in detail [14, 18]. Cells were irradiated with ^4^He ions (LET 123 keV/μm) from a 5.5 MV Singletron accelerator, using the track segment facility at the Radiological Research Accelerator Facility of Columbia University. Unirradiated controls were sham-irradiated alongside radiation-exposed dishes. For chromosomal analyses, mouse embryonic stem cells were irradiated with 1 Gy α particles and dishes were returned to the cell culture incubator for 24 hours, following which, irradiated (6 μm Mylar) and bystander (34 μm Mylar) cell populations were separated and re-seeded into T25 flasks. Chromosome preparations were made at 7 days post-irradiation, slides were blind-coded prior to scoring and metaphases were analyzed for gross chromatid (breaks and gaps on only one arm of a replicated chromosome) and chromosome-type (acentric fragments and rings as well as dicentrics when detected) aberrations using Giemsa staining [19].

### H1299 cell irradiation and micronucleus assay

Irradiation of cells and detection of micronuclei were performed as published [14, 18], H1299 and H1299*shRAD9* cells (1 × 10^6^) were plated onto concentric Mylar dishes a day before irradiation to ensure confluence at the time of treatment. Immediately prior to irradiation, cell culture medium was replaced with fresh medium to remove dead cells. Irradiations were carried out as described above, using a dose of 1 Gy α particles. For each set of experiments, three to five dishes served as unirradiated controls. After irradiation, cells were incubated at 37°C for 4 hours. Cells from directly irradiated (6 μm Mylar) and corresponding bystander (34 μm Mylar) dishes were processed for scoring micronuclei (MN) and for RNA isolation. In brief, dishes were separated, and cells were removed from a small area (≅4 mm^2^) of each Mylar surface separately using trypsin. Cells from the rest of the Mylar were resuspended in lysis solution (miRCURY RNA isolation kit from Exiqon) and stored at −80°C. Trypsinized cells were plated onto four-well chamber slides, and incubated for an additional 17 hours. Growth medium was replaced with fresh medium containing 2 μg/ml cytochalasin B, and cells were incubated for another 26 hours to enrich for those that are binucleated [18]. Cells were fixed for 15 minutes with methanol: acetic acid (3:1), followed by two washes with distilled water. After air drying, slides were briefly stained with SYBR® Green solution (Molecular Probes), cells were visualized with a fluorescence microscope, and a minimum of 1000 binucleated cells were scored per sample. MN percentage was calculated as the number of binucleate cells with micronuclei relative to the total number of binucleate cells in the population examined.

### Microarray and qPCR analyses

RNA was isolated from H1299 cells (miRCURY RNA isolation from Exiqon) with an additional on-column DNase treatment step to eliminate genomic DNA contamination in RNA preparations. RNA quality was assessed using the NanoDrop ND-1000 Spectrophotometer (Thermo Scientific) and RINs were assayed using the Agilent Bioanalyzer (Agilent Technologies), RNA with RINs greater than 8.5 were used for hybridizations. We analyzed n = 5 RNA samples from each condition by microarray hybridization. Cyanine-3 (Cy3) labeled cRNA was prepared from 0.2 μg total RNA using the One-Color Low RNA Input Linear Amplification PLUS kit (Agilent Technologies). Dye incorporation and cRNA yield were monitored with the NanoDrop ND-1000 Spectrophotometer (Thermo Scientific). cRNA (1.6 μg , >9 pmol Cy3 per μg cRNA) was fragmented, hybridized to Agilent Whole Human Genome Oligo 4X44K v2 Microarrays (G4845A) using the Gene Expression Hybridization Kit, and washed following recommendations from Agilent. Slides were scanned with the Agilent DNA Microarray Scanner (G2505B). Default parameters of Feature Extraction Software GE1_1105_Oct12 (Agilent) and grid version 026652_D_F_20120130 were used for image analysis, data extraction, background correction, and flagging of non-uniform features.

Data were exported as text tab delimited files, collated and analyzed using BRB-Array Tools ver. 4.3.2 [20]. Background corrected intensities were log_2_ transformed and median normalized; probes were averaged over replicates and then filtered. Non-uniform outliers or features not significantly above background intensity in 25% or more of the hybridizations were filtered out, leaving 15,859 features. A further filter requiring a minimum 1.3 fold change in at least 20% of the hybridizations was then applied, yielding a final set of 9502 features that were used for subsequent analyses. The microarray data are available through the Gene Expression Omnibus database using accession number GSE55869. Class comparisons were made between paired sample sets of unirradiated controls, directly irradiated and bystander H1299 cells with inherent or reduced RAD9 levels. The choice of samples was based on the percentage of binucleated cells with micronuclei: 1) unirradiated (micronuclei <3%); 2) bystander positive (BP; micronuclei >20%); 3) directly irradiated corresponding to bystander positive (DBP; micronuclei >50%); 4) bystander negative (BN; micronuclei <3%), and 5) directly irradiated corresponding to bystander negative (DBN; micronuclei >50%). Five independent samples for each of the groups were selected for microarray and qPCR studies.

BRB-Array Tools was employed to identify genes differentially expressed in various class comparisons using a random-variance paired t-test, which improves on the standard t-test by sharing information about within-class variation among genes, but which does not require the assumption that all genes have the same variance [21]. The test compares differences in mean log-intensities between classes relative to the expected variation in mean differences computed from the independent samples. Genes with p values less than 0.006 were considered statistically significant. The false discovery rate (FDR) was also estimated for each gene using the method of Benjamini and Hochberg [22] to control for false positives.

The High-Capacity cDNA Archive Kit (Life Technologies, Foster City, CA) was used to prepare cDNA from total RNA. Real time qPCR was performed for selected genes using Taqman assays (Additional file 1). Genes were chosen for this analysis on the basis of differential expression and low FDR, and the results used to confirm microarray experiment findings for the selected genes. For gene expression validation studies, 10 ng cDNA was used as input for replicate reactions. Quantitative real time PCR reactions were performed with the ABI 7900 Real Time PCR System using Universal PCR Master Mix (Life Technologies), with initial activation at 50°C for 120 seconds and 95°C for 10 minutes, followed by 40 cycles of 95°C for 15 seconds and 60°C for 60 seconds. Relative fold-induction was calculated by the ΔΔC_T_ method [23], using SDS version 2.3 software (Life Technologies). Data were normalized to *ACTB* gene expression levels (raw Ct values are included in Additional file 1).

### Ontology and network analysis

The genes responding significantly (p < 0.006 and FDR < 10%) were imported into DAVID, the database for annotation, visualization and integrated discovery (http://david.abcc.ncifcrf.gov/home.jsp). These genes were mapped to DAVID identifiers, and then functionally annotated using DAVID biological processes and molecular function categories. Genes in each functional classification category were compared against those from the NCBI human genome in that category. The one-tailed Fisher exact t-test probability value was used to statistically determine over- or under- representation of classification categories, Bonferroni corrected p values or EASE adjusted Fisher exact p values less than 0.05 were considered significant [24, 25].

The sets of genes significantly differentially regulated in all conditions (FDR < 10%) were imported into Ingenuity Pathways Analysis (IPA; Ingenuity® Systems, http://www.ingenuity.com) to analyze network interactions between them. The imported genes were mapped onto a global molecular network developed from information contained in the Ingenuity Pathways Knowledge Base. Biological functions most significant to these networks were determined, and Fischer’s exact test was used to calculate p values assessing the probability that each biological function assigned to a network was due to chance alone. IPA canonical pathways most significant within the differentially expressed gene sets were also identified. These analyses use curated information on the published relationships between gene products to predict network information. Transcription factor analysis specifically uses information about the relationship between the activity of potential upstream regulatory factors and mRNA abundance changes of target genes for predicting which regulatory factors may be activated or inhibited, based on number of targets and their expression changes. IPA generates a z-score for each factor and uses a cutoff of z > 2 to predict activation and z < −2 to predict inhibition.

## Results

### Impact of *Mrad9*status on delayed chromatid and chromosome aberration formation in direct and bystander irradiated cells

**Figure 1:**
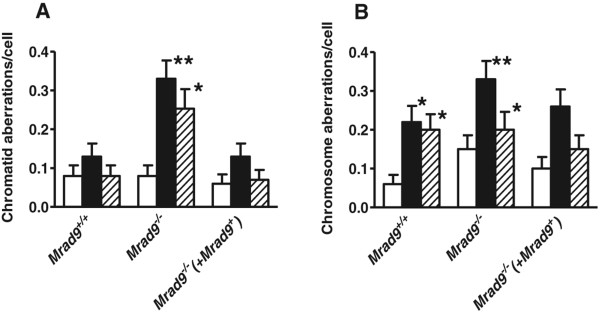
**Impact of** ***Mrad9*** **status on formation of chromatid and chromosome aberrations.** Chromatid **(A)** and chromosome **(B)** aberration frequencies in unirradiated control (open bars), irradiated (closed bars) and bystander (stippled bars) mouse embryonic stem cells as a function of *Mrad9* status (*Mrad9* ^*+/+*^ *, Mrad9* ^*−/−*^ or the latter ectopically expressing *Mrad9* ^*+*^[16]) in mouse embryonic stem cells at 7 days post irradiation (mean ± SD; n = 2). Asterisk and double asterisk depict values that are statistically significant, p < 0.1 and p < 0.05, respectively, between the controls and the corresponding experimental groups. Results from two experiments were pooled and are expressed as mean ± SD. Differences in these data were analyzed using Student’s t-test. (Additional file 2 shows representative pictures of metaphase spreads and aberrations scored in this study).

We examined the effect of *Mrad9* status on chromosome and chromatid aberration frequencies in unirradiated or irradiated cells, using mouse embryonic stem cells either *Mrad9*^*+/+*^, *Mrad9*^*−/−*^ or the latter ectopically expressing *Mrad9*^*+*^ [16]. Representative examples of these aberrations are depicted in Additional file 2. There were no differences in chromatid aberration yields in unirradiated controls regardless of *Mrad9* status (Figure 1A, open bars). However, there were significant differences with respect to induction of chromatid aberrations following exposure to α particles. In contrast to *Mrad9*^+/+^ cells, directly irradiated *Mrad9*^−/−^ cells showed a 4-fold increase in chromatid aberrations at seven days post-irradiation, while bystanders demonstrated a 3-fold increase in chromatid aberrations relative to corresponding unirradiated controls. Ectopic expression of mouse *Mrad9*^+^ in *Mrad9*^*−/−*^ ES cells lowered radiation-induced chromatid aberration frequency levels to those observed in *Mrad9*^+/+^ cells.

Spontaneous chromosome aberrations were higher in *Mrad9*^−/−^ cells, compared with *Mrad9*^+/+^ cells or the mutant ectopically expressing the wild-type gene (Figure 1B). Directly irradiated cells regardless of *Mrad9* status had equivalent increases in chromosome-type aberration frequencies above spontaneous background levels. These aberrations were likely induced directly by irradiation and were in the process of being cleared from the populations, as scoring was performed seven days post-treatment. Chromosome aberration frequencies in *Mrad9*^*+/+*^ bystander cells, but not in *Mrad9*^*−/−*^ or the latter expressing *Mrad9*^*+*^, were elevated above background relative to corresponding unirradiated control populations.

### Reduction of RAD9 expression in H1299 cells enhances induction of micronuclei by direct and bystander radiation exposure

**Figure 2:**
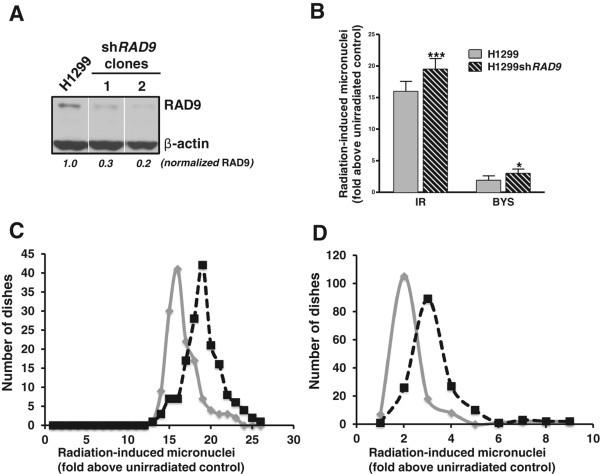
**Effect of RAD9 status on micronuclei formation in H1299 cells A.** Western blot of RAD9 protein in H1299 cells. Lane 1, untransfected H1299 cells, lanes 2 and 3, two stable clones transfected with *shRAD9*. Western blot analysis was performed using RAD9 and β-actin antibodies. Image J was used to determine the level of RAD9 protein after knockdown and the abundance of RAD9 indicates expression normalized to untransfected control H1299. **B**. IR-Induced Micronuclei (MN): micronucleus formation in H1299 (grey bars) and H1299*shRAD9* (striped bars) cells after direct (IR) or bystander (BYS) exposure to 1 Gy of α particles. Data were pooled from five independent sets of experiments each having n ≈ 35 and results represent mean ± SD. Single asterisk indicates a significance of p < 0.05 and triple asterisk, a p < 0.001. **C**. MN fold induction values plotted as a function of number of dishes with each fold change for directly irradiated cells (IR). H1299 (grey line) and H1299*shRAD9* (dashed black line). **D**. same as C, except bystander cells (BYS) were assessed.

In our previous studies, we observed a 2–3 fold increase in radiation induced bystander apoptosis and micronuclei formation in *Mrad9*^*−/−*^ mouse ES cells, compared to the *Mrad9*^*+/+*^ control population [14]. In addition, mutant cells showed higher spontaneous levels of micronuclei, relative to the wild-type control. We extended these studies to human non-small cell lung carcinoma cells, H1299, and two independent stable transfectants expressing shRNA against *RAD9* (H1299*shRAD9*), wherein the corresponding protein levels were reduced 70-80% relative to the untransfected control (Figure 2A). The two transfectants were used interchangeably with no difference in results. In this study, we used 440 dishes (200 for H1299 and 240 for H1299*shRAD9*). Out of these 440, 365 were irradiated dishes and the other 75 served as unirradiated controls (37 for H1299 and 38 for H1299*shRAD9*). Unirradiated H1299 cells showed 3.44 ± 0.82 (mean ± SD) percent micronuclei in binucleated cell populations and H1299*shRAD9* cells had a value of 2.92 ± 0.61 (mean ± SD) percent. Within each track segment experiment (approximately 35 dishes were used in each set of experiments), we normalized the average MN percentage for each bystander and irradiated sample relative to the average for the corresponding unirradiated controls. The normalized value is expressed as MN fold above unirradiated control. An increase in MN fold was observed for both bystander and directly irradiated samples of H1299*shRAD9* and H1299 with inherent levels of RAD9 protein (Figure 2B). Bystander and directly irradiated populations with reduced levels of RAD9, compared with inherent RAD9 counterparts, had a significant elevation in fold induction of MN above corresponding unirradiated controls. Plots of number of dishes with varying MN levels above background were used to assess differences in irradiated and bystander cells before and after RAD9 reduction. By comparing H1299 and H1299*shRAD9* cells, either directly irradiated or bystanders, we found an increase reflected as a modal shift in the number of dishes demonstrating the highest MN fold induction above unirradiated controls in H1299 cells with reduced RAD9. A two-tailed test, using the Z statistic and a 1% level of significance (p value) was performed to check the statistical significance of this finding. As both samples are large (n ≥ 30), we used Z test as opposed to t-test. We rejected the null hypothesis (H_0_) in both bystander and irradiated populations as the Z value is greater than 2.575 (7.159 in bystander and 18.937 in directly irradiated cells). Therefore, the observed difference in the induced number of micronuclei between H1299 and H1299*shRAD9* cells after either bystander or direct irradiation is statistically significant.

### Gene expression profiling in H1299 cells with inherent or reduced levels of RAD9 protein

Microarray gene expression analyses were performed to assess the impact of RAD9 protein reduction on H1299 cells at the molecular level in the absence of radiation. RNA from H1299 cells, untransfected or stably transfected with *RAD9* shRNA and demonstrating a reduction in corresponding protein abundance (Figure 2A), were hybridized to Agilent Human whole genome arrays. BRB-Array Tools was used for data analysis [20]. A total of 9502 genes, comprising the filtered gene set, were analyzed in a class comparison between unirradiated H1299*shRAD9* and H1299, which revealed 1845 genes differentially expressed between these two classes (Additional file 3). Of these, 1112 genes (60%) were down regulated and 733 (40%) were up regulated. We analyzed these genes using DAVID functional annotation and looked for categories enriched after reduction of *RAD9* in H1299 cells. Using an EASE score of 0.1 as a universal cut-off, we found that down regulated genes were enriched for inter- and intra-cellular functions, such as cytoskeletal and actin binding (p value <10^−4^), GTPase activity (p value 0.0009), protein kinase inhibitor activity (p value 0.002), cyclin-dependent kinase regulatory activity (p value 0.01) and other broad categories of enzymatic activity, such as transferase, carbohydrate kinase and oxidoreductase activity (Table 1A). Performing a similar functional annotation on the 733 upregulated genes revealed enrichment of processes such as RNA methyl-transferase activity (p value 0.002), DNA binding activity (p value 0.01) and even transcription factor activity (p value 0.04), which suggested that RAD9 reduction in H1299 cells affects multiple levels of cellular function (Table 1B). We also investigated important regulatory pathways using both DAVID functional annotation and IPA networks, which suggested that two signaling mechanisms, Interferon signaling via STAT1 (p value 0.0006) and Interleukin-4 signaling via STAT6 (p value 0.01), were significantly altered at the RNA level.

**Table 1:** **Enriched GO categories among H1299** ***shRAD9*** **vs. H1299 differentially expressed genes**

**A. H1299** ***shRAD9*** **vs. H1299, down regulated genes**		
**Gene Ontology Term (molecular functions)**	**Gene Count**	**p value**
**Cytoskeletal protein binding**	49	0.00001
**Actin binding**	33	0.0002
**GTPase regulator activity**	36	0.0009
**Growth hormone receptor binding**	3	0.001
**Phospholipid binding**	19	0.002
**Phospho-inositide binding**	13	0.002
**Protein kinase inhibitor activity**	7	0.002
**Kinase inhibitor activity**	7	0.002
**GTPase binding**	13	0.005
**Protein kinase regulator activity**	10	0.008
**PIP-3,4,5-trisphosphate binding**	3	0.009
**Cyclin-dependent protein kinase**	4	0.01
**Microtubule binding**	9	0.01
**NAD binding**	3	0.01
**Kinase activity**	58	0.02
**Oxidoreductase activity**	12	0.02
**Tubulin binding**	11	0.02
**Carbohydrate kinase activity**	4	0.02
**Phospho-transferase activity**	51	0.02
**Glutathione transferase activity**	4	0.02
**Transferase activity**	63	0.03
**De-oxyribonuclease activity**	5	0.03
**Guanyl-nucleotide exchange factor activity**	14	0.03
**Rab GTPase activator activity**	6	0.04
**Heparin binding**	10	0.04
**B. H1299** ***shRAD9*** **vs. H1299, up regulated genes**		
**Gene Ontology Term (molecular functions)**	**Gene Count**	**p value**
**Nor-epinephrine binding**	2	0.002
**RNA methyl-transferase activity**	4	0.002
**Myosin binding**	4	0.002
**Structure-specific DNA binding**	9	0.01
**Single-stranded DNA binding**	5	0.01
**Glycoprotein binding**	4	0.01
**Coenzyme binding**	9	0.03
**Transcription factor activity**	33	0.04

### Gene expression profiling in direct and bystander irradiated H1299 cells with reduced levels of RAD9 protein

Gene expression was analyzed in H1299 cells with or without *shRAD9*, after direct and bystander exposure to α particles. Five independent samples for each group, namely, unirradiated controls, directly irradiated samples and corresponding bystander samples were selected for microarray and qPCR experiments. The choice of samples was based on micronuclei levels in binucleated cells as described above in the Materials and Methods. Micronuclei formation was assessed initially in cells on a small portion of the Mylar dish, with the remainder harvested for RNA isolation at 4 hours after irradiation. Control, directly irradiated and bystander sample RNA were hybridized to Agilent Human whole genome arrays. Using the class comparison tool of BRB-Array Tools [20], we identified genes with significant differential expression in direct and bystander cells compared with unirradiated controls. We found no significant differentially expressed genes in the H1299 parental cells at 4 hours after irradiation. However, in H1299*shRAD9* cells, there was a significant response at the transcriptomic level in both directly irradiated and bystander cells.

We consider those bystanders that showed a micronucleus percentage in binucleates of >20%, successful in bystander signal transmission from the corresponding irradiated cells, and we call these cell populations, bystander positive (BP). An unsuccessful transmission would be when the irradiated cells were unable to induce a micronucleus response in the neighboring bystander cells (micronucleus index < 3%), which we call “bystander negative (BN)”. In directly irradiated cells that successfully transmitted a bystander response (direct bystander positive; DBP), 572 genes were differentially expressed (p < 0.006 and false discovery rate, FDR < 10%; Additional file 4). In the corresponding bystander positive (BP; micronucleus index >20%) cells, 254 genes were differentially expressed (p < 0.006 and FDR < 10%; Additional file 5). There were 146 genes common to both directly irradiated and bystander positive gene sets.

Next, we compared H1299*shRAD9* directly irradiated cells unable to transmit a bystander signal (direct bystander negative; DBN) and their matched bystander negative (BN; micronucleus index <3%) cells to their matched H1299*shRAD9* unirradiated controls. We found 591 significantly differentially expressed genes (p < 0.006 and FDR < 10%, Additional file 6) in DBN and only 3 genes differentially expressed (p < 0.006 and FDR < 10%, Additional file 7) in the BN condition, respectively. The lack of a transcriptional response in the bystander negative population corresponded to the lack of micronuclei induction. Comparing gene expression changes in directly irradiated cells corresponding to bystander positive (DBP) and directly irradiated cells corresponding to bystander negative (DBN); we found 311 responding genes that overlapped between these two groups, representing approximately half the genes in each class.

We analyzed the differentially expressed genes revealed by the microarray studies for enrichment of biological processes, using the DAVID functional annotation database. In H1299*shRAD9* directly irradiated cells corresponding to bystander positive (DBP), 572 differentially expressed genes were used for analysis. Functional annotation revealed that GO biological terms cell cycle (p value 10^−10^), M phase of mitosis (p value <10^−9^), and cell division (p value 10^−8^) were significantly enriched in this gene set (Table 2). Out of the 254 genes significantly changed in H1299*shRAD9* bystander positive cells, ontology analysis showed that the biological processes RNA metabolism (p value 0.05) and cell organization (p value 0.02) were mostly affected in bystander cells. Cell cycle processes and cell division were not affected in the bystander population; however, processes such as cell re-organization were significantly enriched in both gene sets (p value of <0.02) (Table 2).

**Table 2:** **Enriched GO categories among differentially expressed genes in directly irradiated (DBP) vs. controls and bystander positive (BP) vs. controls**

Gene Ontology Term (Biological process)	DBP (p value)	BP (p value)
**Organelle fission**	3.3 × 10^−11^	NS^a^
**Cell cycle phase**	5.7 × 10^−10^	NS^a^
**Nuclear division**	8.1 × 10^−10^	NS^a^
**M phase of mitotic cell cycle**	1.3 × 10^−9^	NS^a^
**Cell division**	3.8 × 10^−8^	NS^a^
**Cell organization**	0.00001	0.02
**Cellular process**	0.00004	0.0003
**Microtubule-based process**	0.0002	NS^a^
**Cellular component organization**	0.001	NS^a^
**Macromolecule metabolic process**	NS^a^	0.0015
**Chromosome segregation**	0.002	NS^a^
**RNA metabolic process**	NS^a^	0.05

^a^NS = not significant (p > 0.05).

### Quantitative PCR validation of microarray changes

**Figure 3:**
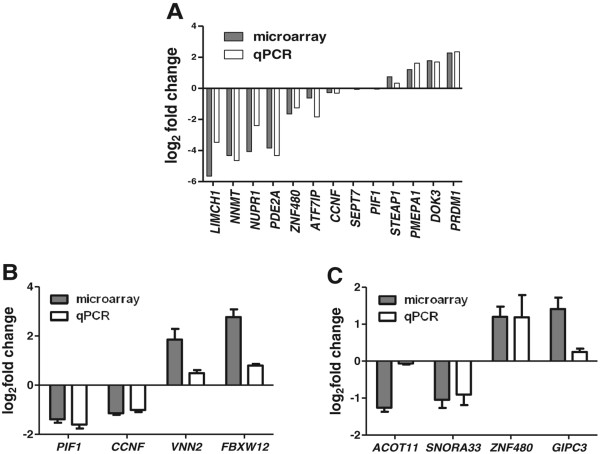
**Comparison of gene expression assessed by microarray and real time qPCR.** Relative gene expression values are log_2_ (fold change) compared to matched controls. Each histogram represents the mean of 5 biological replicates with SEM error bars, where applicable. Grey bars are microarray measurements and open bars are from real time quantitative PCR. **A**. Comparison of gene expression in unpaired H1299 and H1299*shRAD9* samples. **B**. Comparison between directly irradiated cells corresponding to bystander positive (DBP) with unirradiated controls and **C**. Comparison between bystander positive (BP) cells with unirradiated controls. Genes monitored are listed on the x-axis.

We selected genes for qPCR validation on the basis of expression fold change and low FDR. We selected 5 of the most up regulated (*PRDM1, DOK3, CD86, ADCY7,* and *STAT6*) and 5 of the most down regulated (*LIMCH1, NNMT, NUPR, FTCD* and *PDE2A*) genes after RAD9 reduction in H1299. Log_2_ transformed fold changes ranged from −5.6 to +2.3 by microarray analysis. We also validated by qPCR genes that did not show significant changes in mRNA levels by microarray in RAD9-reduced H1299 (*ATF7IP*, *CCNF*, *SEPT7* and *PIF1*), and other genes that were selected based on fold change alone (*ZNF480*, *STEAP1* and *PMEPA1*). We found a close correlation between the measured fold change for 13 out of these 17 genes by microarray and qPCR (Figure 3A).

To verify gene expression changes detected by microarray analyses after direct irradiation, we selected two genes with the highest fold change (*FBXW12* and *VNN2*) and two with the lowest fold change (*PIF1* and *CCNF*) in H1299*shRAD9* directly irradiated corresponding to bystander positive (DBP) cells, for qPCR validation. From H1299*shRAD9* bystander positive (BP) cells, we selected two genes with the highest fold change (*GIPC3* and *ZNF480*) and two with the lowest fold change (*ACOT11* and *SNORA33*). For each condition, mRNA changes for two out of four genes were almost identical as measured by microarray and qPCR (Figure 3B and C). All the genes that were validated also demonstrated a close correlation between microarray and qPCR measurements across other class comparisons (data not shown).

### Comparative gene network analysis in H1299 cells containing reduced or inherent levels of RAD9 protein

**Figure 4:**
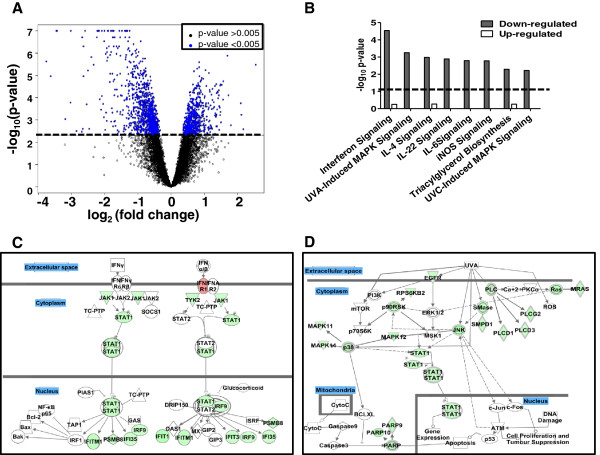
**Network analysis and comparison of gene regulation in H1299** ***shRAD9*** **cells relative to H1299 cells with inherent levels of RAD9. A**. Volcano plot showing genes whose expression is significantly altered (blue), when RAD9 is reduced, above the p value cut-off -log_10_ (0.005) shown as the bold dashed line; log_2_ (fold change) on the x-axis and -log_10_ (p value) on the y-axis. **B**. Pathways affected after RAD9 reduction in H1299 cells: pathways were ordered by decreasing significance [−log10 (p value)] in down regulated genes (solid grey bars). Up regulated genes (open bars) did not show pathway enrichment. **C**. Interferon signaling pathway and **D**. UVA-MAPK signaling pathway were top pathways affected at various levels of signal transduction after RAD9 reduction in H1299 cells. The IPA canonical pathways are shown overlaid with changes of gene expression indicated by different colors. Up regulated genes are red and down regulated genes green, blank nodes are genes that are in the pathway but not in the dataset. Solid lines indicate direct interactions between molecules and dashed lines indicate indirect interactions. Bold grey lines separate cellular compartments, such as extracellular space, cytoplasm, mitochondria and the nucleus. In the UVA-MAPK pathway diagram, signal transduction affects biological processes, such as apoptosis and cell proliferation downstream of nuclear gene expression changes.

The 1845 genes differentially regulated after *shRAD9* mediated reduction and their corresponding expression fold changes were uploaded into the Ingenuity Pathway Analysis (IPA) suite. Of these differentially expressed genes, 65% (1016 identified in IPA) were down regulated. We next queried well known pathways predicted to be affected in these cells, given the large number of genes altered in expression by RAD9 reduction (Figure 4A). The top pathway selected by IPA was Interferon signaling (p value 10^−5^) (Figure 4B), in which mRNA changes occur in genes at all levels of this network (Figure 4C), with an overall reduction in mRNA of multiple known target genes, such as *IRF9*, *IFITM1*, *PSMB8* and *IFI35* [26]. We also looked for other well-defined pathways that were subject to a similar suppression at the mRNA level, by exploring the information and networks from down regulated genes only (65% of the total identified genes affected). The UVA-induced MAPK signaling pathway was significantly repressed after RAD9 reduction (p value 10^−3^) (Figure 4B and D), where repression of multiple transcripts encoding members of regulatory complexes; p38MAPK, JNK and PARP was observed. In both pathways, STAT1 (mRNA down regulated −1.6 fold) appeared as a central player involved in signal transduction. This suggests interference in the stress response of cells following RAD9 reduction, possibly compounded in H1299 cells by the lack of p53 and its downstream effectors. A similar analysis of up regulated genes did not reveal a significant effect on other pathways in IPA (Figure 4B).

### Comparative gene network analysis in direct and bystander irradiated H1299 cells with reduced levels of RAD9 protein

**Figure 5:**
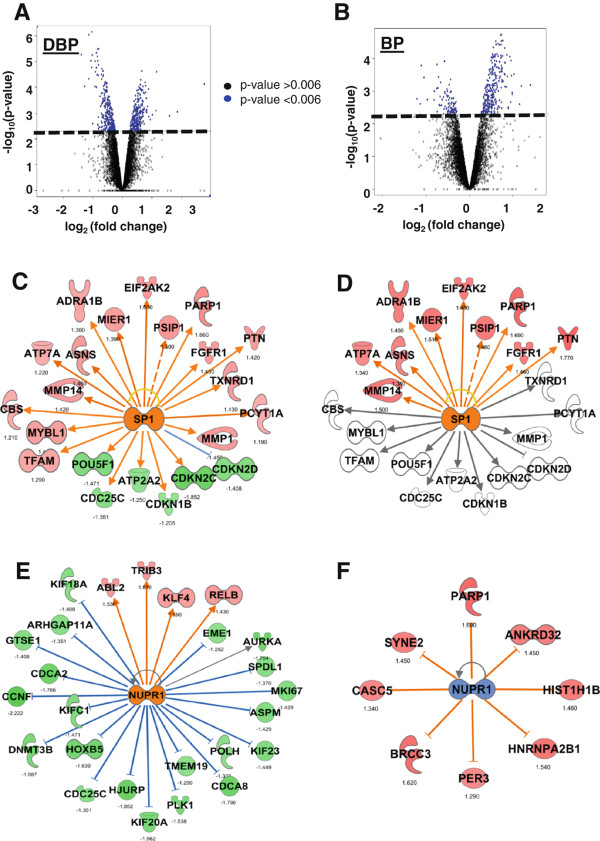
**Network analysis and comparison of regulation in H1299** ***shRAD9*** **bystander response. A** and **B**. Volcano plots showing significantly responding genes (blue) above the p value cut-off -log_10_ (0.006) shown as the bold dashed line in directly irradiated cells corresponding to bystander positive (DBP) and bystander positive (BP) H1299*shRAD9* cells, respectively. Values are log_2_ (fold change) on the x-axis and -log_10_ (p value) on the y-axis. **C**. and **D**. Ingenuity Pathways analysis (IPA) generated SP1 network, overlaid with relative gene expression in directly irradiated (DBP) and corresponding bystander positive (BP) cells, respectively. **E**. Ingenuity Pathways analysis (IPA) generated NUPR1 networks, overlaid with relative gene expression in directly irradiated cells corresponding to bystander positive (DBP). **F**. IPA generated NUPR1 network in bystander positive (BP) cells. In all network diagrams, up regulated genes are colored red and down regulated genes green, with fold changes shown under the nodes. Blank nodes are genes that are in the network but not in the dataset. Solid edges (lines and arrows between nodes) represent direct interactions between molecules and dashed edges represent indirect associations between molecules based on information in the Ingenuity knowledge base. Orange colored lines and nodes represent known activation and blue colored lines and nodes represent known inhibition. A grey line represents an unchanged or unpredicted relationship between two nodes. Numbers under nodes are fold-change values.

We compared gene expression networks generated from differentially expressed genes in the H1299*shRAD9* directly irradiated cells corresponding to bystander positive (DBP; 572 significantly differentially regulated genes) and in corresponding bystander positive cells (BP; 254 significantly differentially regulated genes). Figure 5A shows the volcano plot of expression changes and corresponding p values in DBP cells, where 50% of the 572 genes were up regulated (all blue dots above the dashed line are statistically significant genes with p values <0.006). Figure 5B is the volcano plot of expression changes and corresponding p values in BP cells, where 85% of the 254 genes were up regulated (all blue dots above the dashed line are statistically significant genes with p values <0.006). For network analyses, we used the IPA core analysis tool, which compares two or more sets of genes and their corresponding fold changes based on networks and prediction of regulatory mechanisms. Comparison of networks within these two cell populations revealed both similarities and differences in gene regulation. This has been documented previously in other bystander gene expression studies [6, 9]. IPA upstream regulator analysis predicted activation of the SP1 transcription factor (z-score +2.61 in DBP cells; +2.5 in BP cells) (Figures 5C and D) after direct and bystander radiation exposure in H1299*shRAD9*. Although this network was also predicted to be activated in H1299*shRAD9* directly irradiated corresponding to bystander negative (z-score +2.45 in DBN cells), 10 out of 22 genes differentially expressed in DBP, shown in the SP1 network in Figure 5C, were not similarly affected in DBN cells.NUPR1 DNA binding nuclear phospho-protein p8, which acts as a transcription regulator via chromatin binding, was also predicted to be activated in directly irradiated cells corresponding to bystander positive (z-score +2.61 in DBP), but inactivated in bystander positive cells (z-score −1.89 in BP) (Figures 5E and F). There was no overlap of genes in the NUPR1 networks for these two cell populations, suggesting that regulation of NUPR1 might be different in directly irradiated cells (DBP) and corresponding bystander positive (BP) cells. Of note, 22 out of 25 genes in the NUPR1 network of directly irradiated corresponding to bystander positive (DBP), shown in Figure 5E, overlapped with the NUPR1 network in directly irradiated cells corresponding to bystander negatives (DBN, not shown) and the direction of change was the same in both populations.

**Figure 6:**
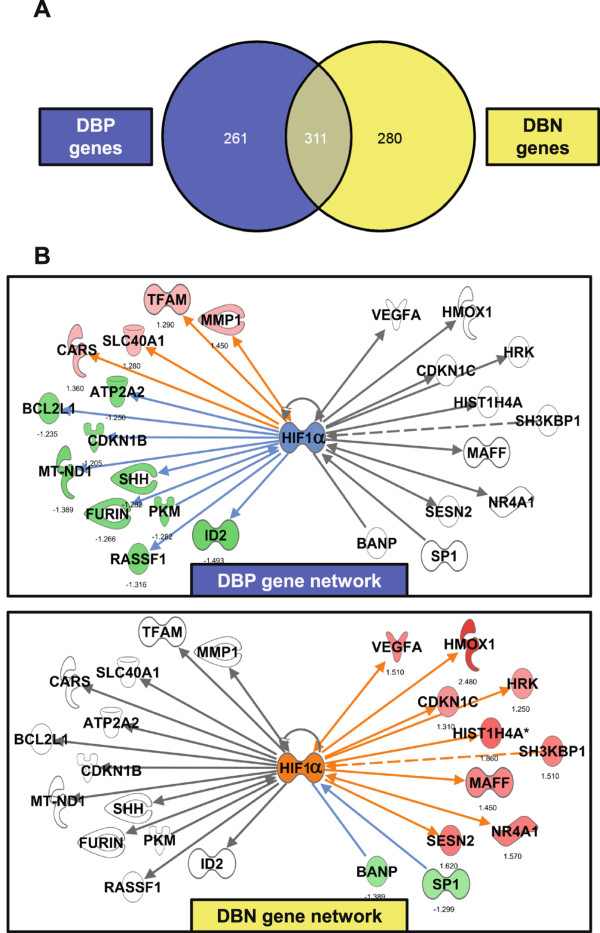
**Network analysis and comparison of regulation in H1299** ***shRAD9*** **cells directly irradiated and corresponding to bystander positive (DBP) or to bystander negative (DBN) cells. A**. Venn diagram of overlap in significantly differentially expressed genes from directly irradiated cells corresponding to bystander positive, DBP and those corresponding to bystander negative, DBN cells, constructed using VENNY. Venn diagrams were generated using Venny [35]. **B**. Ingenuity Pathways analysis (IPA) generated HIF1α network connecting to genes that were only significantly changed in DBP cells, left of the HIF1α node, and genes that were only significantly changed in DBN cells, right of HIF1α node. In the upper panel, genes are overlaid with relative expression levels in directly irradiated cells corresponding to bystander positive (DBP). In the lower panel, genes are overlaid with relative expression in directly irradiated cells corresponding to bystander negative (DBN). Up regulated genes are red and down regulated genes green, with fold changes shown under the nodes. Blank nodes are genes that were in network but not in the dataset used to show fold-changes. Solid edges (lines and arrows between nodes) depict direct interactions between molecules and dashed lines represent indirect associations between molecules based on information in the Ingenuity knowledge base. Orange colored lines and nodes represent known activation and blue colored lines and nodes represent known inhibition. A grey line represents an unchanged or unpredicted relationship between two nodes.

The numbers of genes differentially regulated in the two directly irradiated cell groups, one corresponding to bystander positive (DBP) and the other corresponding to bystander negative (DBN) cells were 572 and 591, respectively, with approximately 50% of the genes in common to both groups (Figure 6A). These 311 overlapping genes showed similar trends in gene expression and none changed in the opposite direction in the two gene sets. We were interested in the genes unique to each condition i.e. 261 in DBP (left side of the Venn diagram in Figure 6A) and 280 in DBN cells (the right side of the Venn diagram) as they may suggest differences in signaling mechanisms responsible for the very different responses observed in the corresponding bystander cells. Network and upstream transcriptional factor analyses in IPA predicted that the top transcriptional regulator, Hypoxia Inducible Factor 1-alpha subunit, HIF1α was activated in directly irradiated cells corresponding to bystander negative (z-score +2.74 in DBN) and inhibited in directly irradiated cells corresponding to bystander positive (z-score −1.55 in DBP). This transcription factor was selected because of correspondence to the highest significance z-score in DBN gene networks. The network centered on HIF1α, as shown in Figure 6B, is generated from these two unique gene subsets. On the left side of the Figure, in both panels, are genes significant only in directly irradiated cells corresponding to bystander positive (13 genes in DBP that connect with HIF1α). On the right side in both panels of Figure 6B are genes significant only in directly irradiated cells corresponding to bystander negative (11 genes in DBN that connect with HIF1α). The upper panel in Figure 6B depicts the predicted HIF1α inhibition status in directly irradiated cells corresponding to bystander positive (DBP) only, with consequent down regulation of target genes in this group [27]. Some genes such as *MMP1* and *TFAM* were expected to be up regulated following inhibition of HIF1α [28, 29]. However, most genes, such as *BCL2L1* and *CDKN1B*, were down regulated, as expected following HIF1α inhibition/degradation [30, 31]. The genes on the right side of this panel remained unchanged in DBP cells. In contrast, the lower panel of Figure 6B depicts predicted HIF1α activation status and the network of gene expression in directly irradiated cells corresponding to bystander negative, DBN cells, which are now shown as regulated on the right side. In this part of Figure 6B, genes to the left were not significantly changed in DBN cells and remain uncolored. However, on the right of this lower panel, the majority of the genes were expected to be induced following the prediction of HIF1α activation in DBN cells, for example *CDKN1C* and *VEGFA* were expected to be induced following HIF1α activation [32, 33]. The previously mentioned *SP1* gene is also seen in this overlay as a down regulated target gene following HIF1α inhibition [34].

## Discussion

It is well established that a DNA damage response can be induced not only in cells exposed directly to radiation but also in neighboring unirradiated bystander cells (reviewed in [3]). Nevertheless, the signaling mechanisms that mediate this non-targeted effect are not well defined. Previously, we studied the effect of *Mrad9* null in mouse embryonic stem cells, where it was established that these cells, relative to the wild-type control, demonstrate enhanced spontaneous and high-LET radiation induced bystander apoptosis and micronucleus formation, with much less of a differential effect on cell killing by direct or bystander α particle exposure [14]. In the present study, we analyzed the formation of delayed chromosome and chromatid aberrations at seven days after 1 Gy α particle exposure. We found that chromatid aberrations were induced at higher levels in *Mrad9*^*−/−*^ cells, compared with the *Mrad9*^*+/+*^ population, and low background levels were restored in the mutant cells when *Mrad9*^*+*^ was ectopically expressed. These results indicate that *Mrad9* plays a role in protecting against long-term genomic instability after irradiation. Examination of these cell populations up to 28 days post-irradiation revealed continuing induction of chromosome aberrations in the *Mrad9*^*−/−*^ cells, and background levels when *Mrad9* was ectopically expressed (data not shown). These findings in mouse ES cells are consistent with those obtained using H1299, indicating that reduced levels of RAD9 enhance many direct and bystander radiation responses.

This study also focused on the role of human RAD9 in the radiation induced bystander response, using a transcriptome profiling approach. RNA interference was used to reduce RAD9 protein levels in the human non-small cell lung carcinoma cell line, H1299 [36]. Micronucleus formation in binucleate cells, as a measure of the damage response, was used as an endpoint. While all cell populations directly irradiated with 1 Gy α particles showed >50% micronuclei frequency, bystander cells, on the other hand, showed a wide range of micronuclei responses, from background levels to a frequency >20%. Because of the heterogeneity of this response, we assessed damage in individual Mylar dishes, as opposed to pooling cells from multiple dishes. We selected only those bystander RNA samples for further processing that showed a robust micronucleus response (>20%), in an attempt to minimize noise within the microarray gene expression results. These high responders were designated bystander positive and constituted ~3% of the total dishes processed individually for each group, H1299 and H1299*shRAD9*. We also selected bystander negative dishes wherein we observed background levels of micronuclei, for further study. For every bystander sample chosen, we isolated RNA from the paired outer dish (directly irradiated), in which micronuclei formation was consistently >50%, and a matched unirradiated control (micronuclei frequency at background, <3%). The goal of this approach was two-fold, to determine the effect of RAD9 on the bystander response and to suggest a mechanism underlying the observed variability in intensity of that response.

We found that RAD9 reduction had a significant effect on the mean micronuclei fold induction above background in directly irradiated or bystander cells (Figure 2B); reduction of RAD9 also caused a modal shift in the number of dishes having a higher fold of micronuclei above background (Figure 2C and D). This indicates that RAD9 protects against the formation of micronuclei after α particle exposure.

Gene expression was assessed via whole genome microarray analysis of transcripts, initially in unirradiated H1299 and H1299*shRAD9* cells. Differential expression of 1845 genes was detected, and 60% were down regulated in the H1299*shRAD9* cells relative to the H1299 parental control. Previously, it was reported by us that *RAD9* over expression in H1299 cells transcriptionally activated *CDKN1A* [37]. Another study showed that *RAD9* overexpression in H1299 cells initiates a p21(CDKN1A)-dependent senescence program [38]. In the present study, we observed down regulation of *CDKN1A* mRNA (−1.7 fold change, FDR <1.5%) in H1299*shRAD9* cells, consistent with a role for RAD9 in *CDKN1A* transcription control.

Network and ontology analysis of the genes down regulated when RAD9 level is reduced indicated that Interferon signaling and MAPK stress response pathways were suppressed at the mRNA level (Figure 4). Interferons have anti-proliferative properties and promote apoptosis in immune system cells via a STAT1-based mechanism, and the connection between RAD9 reduction and this pathway is not obvious. The STAT1 transcription factor is also central to the UVA-MAPK pathway, which involves p38MAPK and JNK in a signal transduction network leading to expression of target DNA repair genes. There is evidence for the reduction of RAD9 abundance resulting in diminished transcription of nucleotide excision repair genes, such as *DDB2* and *XPC* [39]. However the effect of RAD9 reduction on the UVA-MAPK pathway is a novel finding.

Another interesting result, related to the large number of down regulated genes, was the significance of the cytoskeletal protein binding molecular function category (p value 10^−5^) revealed by gene ontology analyses (Table 1). Enrichment of this functional category after RAD9 reduction was based on 49 down regulated genes related to cell structure. Although the connection between radiation sensitivity and cytoskeletal organization is not well defined, there is evidence that overexpression of cofilin, a protein that promotes disorganization of the actin cytoskeleton, can increase radiation sensitivity of H1299 cells [40]. These findings suggest it would be interesting to study the role of RAD9 protein in mediating a DNA damage response through cell structure organization.

Gene expression was also analyzed four hours after α particle irradiation in bystander and matched directly exposed cells, relative to unirradiated controls. No significantly responding genes were detected in H1299 cells. In another study, H1299 cells were reported to be similarly unresponsive to radiation at the mRNA level, when a subset of radiation response genes were measured after an acute dose of 10 Gy of ionizing radiation [36]. This study looked at gene expression responses up to 24 hours after irradiation and found that H1299 cells did not show a transcriptional response. A study of the gene expression response of the NCI-60 cell line panel, which includes H1299, to 8 Gy of gamma rays, similarly found that p53 played a dominant role in the transcriptional response to radiation [41]. In the absence of p53, E2F transcription factor family member (E2F4) and Retinoblastoma protein family member (RBL2) were predicted as possible regulators of gene expression. In another study on the Lymphoblastoid cell lines TK6 (wild-type p53), NH32 (p53 null) and WTK1 (mutant p53), it was found that in the absence of functional p53, NFκB, E2F1 and E2F4 transcriptional factors dominate the transcriptional response and that the number of genes responding in p53 null cells, over a 24 hour period, was a third of that detected in wild-type p53 cells, with few genes in common [42]. The absence of significant gene expression changes observed in α particle exposed H1299 cells in this study is thus consistent with published reports and likely due to the lack of wild type p53.

We also characterized the transcriptional response of H1299*shRAD9* cells to irradiation. Reduction of RAD9 protein level had a significant effect on gene expression measured four hours after exposure, relative to unirradiated controls. Gene ontology analyses on directly irradiated cells suggested that cell division and cell cycle processes were affected after irradiation (Table 2). IPA analysis of the genes responding to direct irradiation (DBP) and in bystander positive (BP) cells suggested that regulation in DBP and BP cells may be mediated by SP1 and NUPR1 transcription factors, which have not previously been implicated in the bystander response (Figure 5). The SP1 transcription factor, a member of the Retinoblastoma control protein (RCP) family, is known to be a component of the coordinate cellular response after irradiation [43] and can be regulated by ATM-mediated phosphorylation as well as by ATR [44, 45]. Since the 9-1-1 complex is an integral part of ATR signaling, which is also important for the bystander response [15], it is not surprising that SP1 expression is influenced by RAD9 status. SP1 potentially targeted expression of 22 genes in DBP cells, some of which were down regulated [46, 47]. In addition to repression of *CDKN1B* and *CDC25B* mRNA, there was down regulation of *CDKN2* gene transcripts encoding p18-INK4C (CDKN2C) and p19-INK4D (CDKN2D) proteins, which have common functions of binding and inhibiting cyclin/CDK complexes and promoting growth arrest [48]. SP1 also potentially targeted induction of genes promoting cell growth and migration, *MMP1*, *MMP14* and *FGFR1* in directly irradiated cells corresponding to bystander positive, DBP; and *MMP14* and *FGFR1* in bystander positive, BP cells [49–51]. The down regulation of transcripts encoding cell cycle inhibitors observed in DBP cells was not observed in BP cells, implying that irradiation may lead to cell cycle changes in directly hit cells that are not matched bystanders.

Network based transcription factor analysis also predicted NUPR1 to be activated in DBP cells, but repressed in BP cells. NUPR1 is a phospho-protein that inhibits apoptosis and promotes cell growth [52, 53], and is over expressed in cancer cells [54]. Its regulation by phosphorylation leads to gene expression, and a microarray study of NUPR1 silencing revealed changes in gene expression enriched in DNA repair and cell cycle functions [55]. In H1299 directly irradiated cells corresponding to bystander positives, NUPR1 activation was predicted, potentially targeting down regulation of a number of genes after irradiation. This same network was not seen in bystander positive cells, implying alternate regulation of this potential transcription factor.

Bystander negative cells that had background levels of micronuclei also lacked a significant gene expression response. This finding supports the idea that the bystander response is highly variable from dish to dish, with percentages of cell populations demonstrating micronuclei ranging from 1% to 48%. This confirms that our technique of sampling a small portion of the dish for micronuclei formation successfully identified responding and non-responding cell populations.

We used a similar approach as above for comparing the genes that responded differently in the two sets of directly irradiated cells, one corresponding to bystander positive, DBP, and the other corresponding to bystander negative, DBN cells. Prediction analysis suggested HIF1α activation (by protein stabilization) as a potential component that may be involved in DBN cell signaling. The most common stimulus for activation/stabilization of HIF1α is hypoxia, but HIF1α can also be activated by growth factors and ROS [27]. Figure 6 shows the predicted network in which HIF1α may be inhibited/degraded in directly irradiated cells corresponding to bystander positive (DBP; upper panel), and includes some common target genes of the SP1 transcription factor, such as *MMP1* and *CDKN1B*. In directly irradiated cells corresponding to bystander negative, HIF1α was predicted to be activated (DBN; lower panel). HIF1α activation occurs by protein stabilization and dimerization with HIF1α protein followed by translocation to the nucleus and activation of known target genes such as *SESN1* and *VEGFA*. HIF1α activation may also have an effect of suppressing the *SP1* transcript in these cells. SP1 was not predicted to be an active regulator of transcription in directly irradiated cells corresponding to bystander negative, in contrast with the irradiated cells corresponding to bystander positive. There is evidence for a role of HIF1α activation in H1299 cells after radiation and the protein was shown to be activated 3 hours after exposure to 3 Gy of ionizing radiation via a phospho-AKT and phospho-ERK1/2 mechanism involving stabilization by the Hsp90 chaperone [56]. In the same study, HIF1α silencing in H1299 cells promoted radiation resistance and lowered endothelial cell viability via VEGFA in conditioned medium. Further studies will be needed to test the ability of HIF1α to mediate differences in extra-cellular signaling from irradiated cells, and to investigate its potential for influencing or predicting whether or not a bystander response is triggered.

## Conclusions

We have presented evidence that RAD9 plays a role in the radiation induced bystander response and that cells deficient in RAD9 show increased sensitivity to direct and bystander irradiation. We demonstrated heterogeneity in the bystander response, using micronuclei formation as the endpoint. All directly irradiated cell populations had high frequencies of micronuclei, but associated neighboring cells had dramatically different bystander responses. In addition, we provide evidence that HIF1α activation status of irradiated cells may be critical for the ability to elicit a bystander response. We demonstrate further support of a role for RAD9 in the bystander response to irradiation, and identify genetic elements that potentially mediate the response. These results are important as they reveal underlying mechanism and potential targets to modify the cellular response to direct and bystander radiation exposure.

## Electronic supplementary material

Additional file 1: **PCR assay information and Ct values.** (XLS 42 KB)

Additional file 2: **Representative metaphase spreads showing chromosomal breaks.** (PDF 194 KB)

Additional file 3: **Class comparison of genes differentially expressed in H1299** ***shRAD9*** **vs. H1299.** (XLS 1 MB)

Additional file 4: **Class comparison of genes differentially expressed in H1299** ***shRAD9*** **cells 4 hours after direct irradiation (corresponding to bystander positive).** (XLS 358 KB)

Additional file 5: **Class comparison of genes differentially expressed in H1299** ***shRAD9*** **4 hours after irradiation in bystander positive cells.** (XLS 170 KB)

Additional file 6: **Class comparison of genes differentially expressed in H1299** ***shRAD9*** **cells 4 hours after direct irradiation (corresponding to bystander negative).** (XLS 368 KB)

Additional file 7: **Class comparison of genes differentially expressed in H1299** ***shRAD9*** **4 hours after irradiation in bystander negative cells.** (XLS 26 KB)

## Abbreviations

RAD9: Cell cycle checkpoint control protein RAD9
*Mrad9*: Mouse RAD9 homolog gene
NSCLC: Non small cell lung cancer
MN: Micronucleus
IR: Ionizing radiation
GO: Gene ontology
FDR: False discovery rate
IPA: Ingenuity Pathway Analysis
DBP: Direct irradiated corresponding to bystander positive
BP: Bystander positive
DBN: Direct irradiated corresponding to bystander negative
BN: Bystander negative
HIF1α: Hypoxia inducible factor 1-alpha subunit.
