# Supplementary material for: RAD9 deficiency enhances radiation induced bystander DNA damage and transcriptomal response
Source: Radiat Oncol. 2014 Sep 18;9:206. doi: 10.1186/1748-717X-9-206 (PMC4261775; doi:10.1186/1748-717X-9-206)
Supplement: Supplementary file 2 — Additional file 2: Representative metaphase spreads showing chromosomal breaks. (PDF 194 KB) [file 13014_2014_1171_MOESM2_ESM.pdf]

Additional file 2

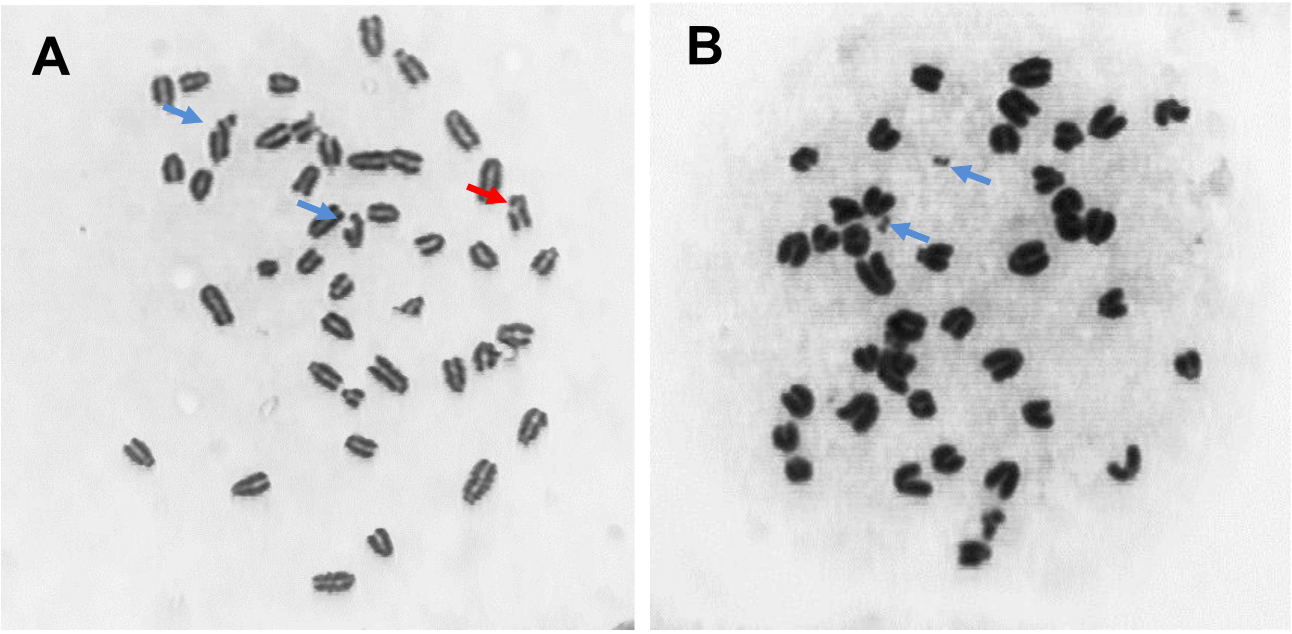

Representative images depicting the types of chromatid (panel A) and chromosome-type (panel B) aberrations scored in this study. In panel A, blue arrows point to chromatid breaks and the red arrow points to a chromatid gap. In panel B, blue arrows point to acentric chromosome fragments.
